# SlSERK3B Promotes Tomato Seedling Growth and Development by Regulating Photosynthetic Capacity

**DOI:** 10.3390/ijms25021336

**Published:** 2024-01-22

**Authors:** Zhiqi Ding, Yandong Yao, Kangding Yao, Xuemei Hou, Zhuohui Zhang, Yi Huang, Chunlei Wang, Weibiao Liao

**Affiliations:** College of Horticulture, Gansu Agricultural University, 1 Yinmen Village, Anning District, Lanzhou 730070, China; 15693198831@163.com (Z.D.); yyd614636237@163.com (Y.Y.); 19119882925@163.com (K.Y.); hxm1511704@163.com (X.H.); 13837951879@163.com (Z.Z.); huangyi202309@163.com (Y.H.); wangchunlei@gsau.edu.cn (C.W.)

**Keywords:** brassinosteroids, *SlSERK3B*, virus-induced gene silencing, pigment content, photosynthetic capacity

## Abstract

Brassinosteroids (BRs) are a group of polyhydroxylated steroids for plant growth and development, regulating numerous physiological and biochemical processes and participating in multi-pathway signaling in plants. 24-Epibrassinolide (EBR) is the most commonly used BR for the investigation of the effects of exogenous steroidal phytohormones on plant physiology. Although *SlSERK3B* is considered a gene involved in the brassinosteroid (BR) signaling pathway, its specific role in plant growth and development has not been reported in detail. In this study, tomato (*Solanum lycopersicum* L.) seedlings treated with 0.05 μmol L^−1^ EBR showed a significant increase in plant height, stem diameter, and fresh weight, demonstrating that BR promotes the growth of tomato seedlings. EBR treatment increased the expression of the BR receptor gene *SlBRI1*, the co-receptor gene *SlSERK3A* and its homologs *SlSERK3B*, and *SlBZR1*. The *SlSERK3B* gene was silenced by TRV-mediated virus-induced gene silencing (VIGS) technology. The results showed that both brassinolide (BL) content and BR synthesis genes were significantly up-regulated in TRV-SlSERK3B-infected seedlings compared to the control seedlings. In contrast, plant height, stem diameter, fresh weight, leaf area and total root length were significantly reduced in silenced plants. These results suggest that silencing *SlSERK3B* may affect BR synthesis and signaling, thereby affecting the growth of tomato seedlings. Furthermore, the photosynthetic capacity of TRV-*SlSERK3B*-infected tomato seedlings was reduced, accompanied by decreased photosynthetic pigment content chlorophyll fluorescence, and photosynthesis parameters. The expression levels of chlorophyll-degrading genes were significantly up-regulated, and carotenoid-synthesising genes were significantly down-regulated in TRV-*SlSERK3B*-infected seedlings. In conclusion, silencing of *SlSERK3B* inhibited BR signaling and reduced photosynthesis in tomato seedlings, and this correlation suggests that *SlSERK3B* may be related to BR signaling and photosynthesis enhancement.

## 1. Introduction

Photosynthesis is one of the most important metabolic process for the growth, development and yield of plants [1]. Meanwhile, crop yields depend directly on the efficiency of the photosynthetic machinery in acquiring light energy [2]. It has been shown that a sustained increase in leaf photosynthesis led to increased yield [3]. By maintaining efficient photosynthesis during the tassel and flowering stages of rice, it could help rice resist cold [4]. Therefore, the factors that regulate photosynthesis play a very important role in plant growth and development.

Brassinosteroids (BRs) are a class of steroid phytohormones with a broad spectrum of biological functions, and are involved in various plant growth and developmental processes such as seed germination, root growth, stem elongation, leaf morphogenesis, stomatal formation, and flower development [5]. Brassinolide (BL), the most active of the BRs, was first isolated from European rape pollen [6]. To date, the BR signaling pathway has been extensively explored through a variety of approaches. BR was sensed at the plasma membrane by the extracellular domains of the Brassinosteroid-Insensitive1 (BRI1) receptor and its co-receptor BRI1-associated receptor kinase 1 (BAK1) [7]. Meanwhile, BAK1 and BRI1 interacted in vitro and in vivo, and the transphosphorylation of BRI1/BAK1 affected the early course of the BR signaling pathway [8]. BAK1 has also been annotated as *AtSERK3* (a member of the *AtSERK* subfamily). It consists of 5 LRR-RLK, named *AtSERK1-5* [9]. In Solanaceous plants, *SERK3* homologs have been characterized from *N. benthamiana*, *N. attenuata* and tomato (*Solanum lycopersicum* L.). In *N. benthamiana*, two *AtSERK3/BAK1* homologs, *NbSERK3A* and *NbSERK3B*, were identified [10]. There is only one homologous gene, *NaBAK1*, in *N. attenuate* [11]. Tomato has three *SlSERK* members. Two of them exhibited particularly high levels of sequence similarity to *AtSERK3* and, therefore, they were named *SlSERK3A* and *SlSERK3B* [9]. In Arabidopsis, compared with wild-type plants, *bak1* gene deletion mutant plants showed reduced sensitivity to BR and diminished BR inhibition of root growth [12,13]. A recent study has revealed an important role of BAK1 /SERK3 in negatively regulating ABA signaling during Arabidopsis seed germination and primary root growth but positively modulating ABA-induced stomatal closure, thus optimizing the plant growth under drought stress [14]. However, it has not yet been revealed how BAK1/SERK3 affects the growth and development of tomato seedlings.

The ability of the exogenous application of BR to increase the photosynthetic rate of plants has been extensively demonstrated in a wide range of plants. However, it is not clear what exactly leads to BR-related changes in photosynthesis and its associated properties. The most popular hypothesis is that BR improves the efficiency of the Calvin cycle of photosynthesis by increasing the levels of the relevant enzymes [15]. BR prevented the loss of photosynthetic pigments by activating or inducing the synthesis of enzymes involved in chlorophyll biosynthesis [16]. Meanwhile, compared with the wild type photochemical quenching (qP), the electron transfer rate (ETR), PSII photochemical maximum quantum yield (Fv/Fm), net photosynthesis rate (PN), and chlorophyll accumulation were significantly reduced in the *altered brassinolide sensitivity1* (*abs1*) mutant [2]. In addition, the application of 24-epibrassinolide (EBR) increased the cold stress tolerance of black pepper by net photosynthetic rate (Anet), Fv/Fm, and qP [17]. However, it remains to be investigated whether genes related to the BR signaling pathway can influence plant photosynthesis.

Several roles of the BR signaling pathway in growing and developing plants have been reported. The BR-signaling downstream suppressor *BIN2* interacted with *SLFRIGIDA-LIKE* to induce early flowering in tomato [18]. The BR signaling component *SlBZR1* was a master regulator of tomato fruit ripening with the potential to improve tomato quality and carotenoid biofortification [5]. However, there are few reports on the key genes in the BR pathway that regulate the growth and development of tomato seedlings. Therefore, it is important to investigate the possible effects of key genes in the BR pathway on the growth and development of tomato seedlings. Tomato is one of the world’s most important horticultural crops [19], with a distinctive flavor and a wealth of nutrients [20]. Meanwhile, tomato is often studied as a model plant because of its diploid genome, relatively small genome (about 950 Mb), well-annotated genome sequence [21], short life cycle, effective transient virus-induced gene silencing, and stable transformation [22]. In this study, we investigated the roles of *SlSERK3B*, a key gene in the BR signaling pathway, in the growth and development of tomato seedlings by TRV-mediated virus-induced gene silencing (VIGS) analysis.

## 2. Results

### 2.1. Exogenous EBR Promotes the Growth and Development of Tomato Seedlings and Up-Regulates the Expression Levels of Key Genes in the BR Signaling Pathway

**Figure 1:**
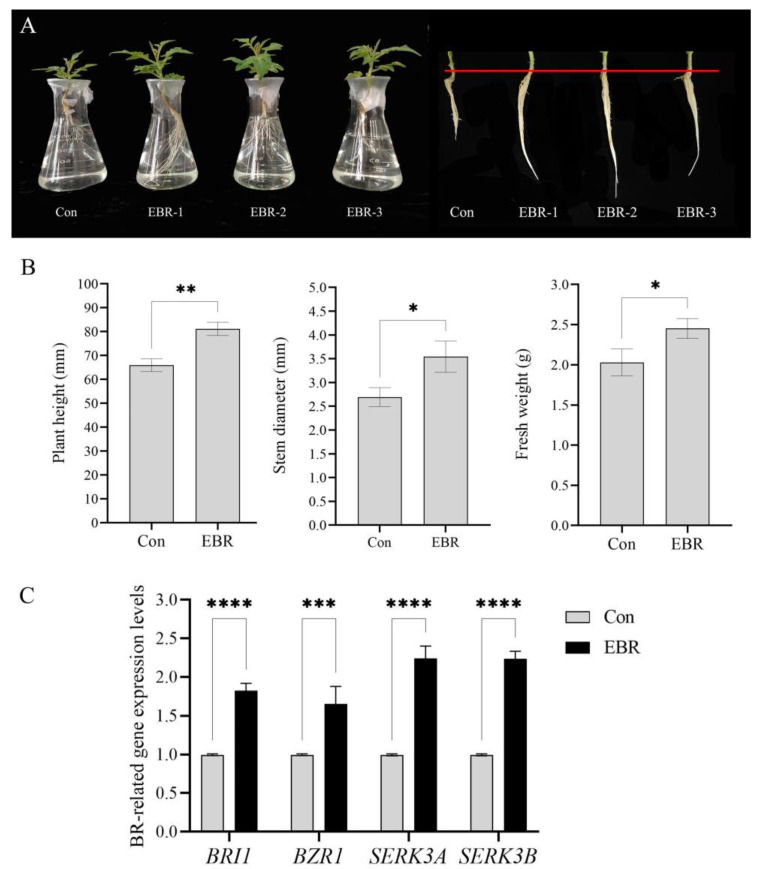
Effect of exogenous EBR treatment on the growth and development of tomato seedlings and the expression of key genes in the BR signaling pathway. (**A**) Phenotype of tomato seedlings. (**B**) Plant height, stem thickness and fresh weight. (**C**) Expression levels of key genes *(BRI1*, *BZR1*, *SERK3B* and *SERK3A*) in the BR signaling pathway. Expression values were normalized to the control for each gene. Bars are means ± SD of three biological replicates. Asterisks indicate significant differences between BR treatment and control groups (* *p* ≤ 0.05, ** *p* ≤ 0.01, *** *p* ≤0.001, **** *p* ≤ 0.0001). The number 1 means standardize the control group, namely, the expression level of control divided by itself.

To elucidate the possible role of BR in the growth and development of tomato seedlings, tomato seedlings were treated with a concentration of 0.05 μmol L^−1^ 24-epibrassinolide (EBR). Compared with the control, the EBR-treated tomato seedlings showed higher appearance in the above-ground and root sections (Figure 1A). EBR treatment significantly increased the plant height, stem diameter, and fresh weight of tomato seedlings compared to the control. EBR treatment increased plant height significantly compared to the control, with a 23.1% increase. Meanwhile, EBR treatment increased stem diameter and fresh weight by 31.6% and 20.7%, respectively, compared to the control. (Figure 1B). These results suggest that BR can promote the growth and development of tomato seedlings.

The expression levels of several key genes in the BR signaling pathway (BR receptor gene *BRI1*, the co-receptor gene *SERK3A* and its homologue *SERK3B*, and the transcription factor *BZR1*) were quantified using quantitative RT-PCR (qPCR) (Figure 1C). Compared with the control, BR treatment up-regulated the expression of *SlBRI1*, *SlBZR1*, *SlSERK3B*, and *SlSERK3A* by 82.3%, 65.6%, 124.3%, and 123.5%, respectively.

### 2.2. SlSERK3B Gene Silencing May Impede BR Signaling

**Figure 2:**
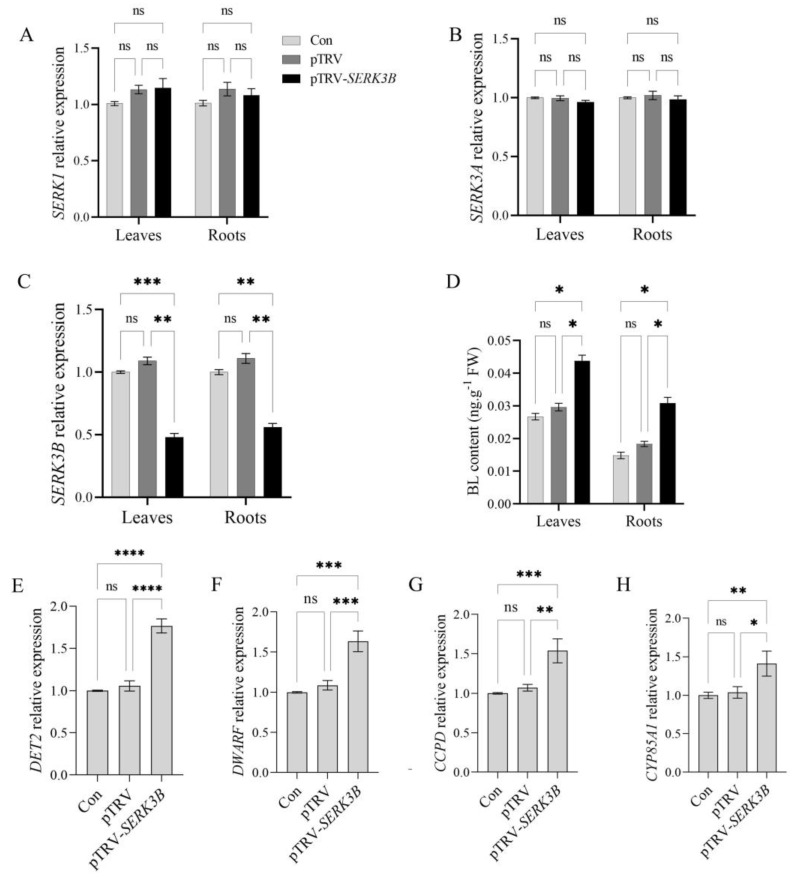
Effect of silencing *SlSERK3B* gene on BL content and expression of its synthesizing genes. (**A**,**B**) Relative expression of *SlSERK1* and *SlSERK3A* gene in leaves and roots; (**C**) relative expression of the *SlSERK3B* gene in leaves and roots; (**D**) endogenous BL content of seedlings in the control, empty TRV-infected and *SlSERK3B*-infected groups; (**E**–**H**) relative expression of BR synthesis genes in control, empty TRV-infected, and *SlSERK3B*-infected seedlings. Expression values were normalized to the control for each gene. Bars are means ± SD of 3 biological replicates. Asterisks indicate significant differences between control, empty TRV-infected as well as *SlSERK3B*-infected seedlings (* *p* ≤ 0.05, ** *p* ≤ 0.01, *** *p* ≤ 0.001, **** *p* ≤ 0.0001, ns: no significant difference).

To verify that the plant phenotype observed in pTRV-*SERK3B* plants was associated with reduced expression levels of *SlSERK3B* and was not caused by the silencing of other *SlSERK* members, the expression of *SlSERK3B* and its homologs *SlSERK1* and *SlSERK3A* in leaves and roots of control, empty TRV-infected, and silenced seedlings were determined using qRT-PCR. Compared with that in empty TRV-infected seedlings, the relative expression of *SlSERK3B* genes in leaves and roots in TRV-*SlSERK3B*-infected seedlings was reduced by 56.3% and 54.9%, respectively; whereas there were no significant differences in the expression of *SlSERK1* or *SlSERK3A* between empty TRV-infected and TRV-*SlSERK3B*-infected seedlings (Figure 2A–C), thereby ensuring that only the target gene was silenced. Meanwhile, the BL content in the leaves and roots of the TRV-*SlSERK3B*-infected seedlings was increased by 47.8% and 68.2%, respectively, compared with that of the empty TRV-infected seedlings (Figure 2C). In addition, the expression levels of the BR synthesis-related genes *CYP85A1*, *CCPD*, *DWARF* and *DET2* were significantly higher in the *SlSERK3B*-infected seedlings than in the control and empty TRV-infected seedlings (Figure 2D–G). However, there were no significant differences in the expression of the *SlSERK3B* gene and BR synthesis genes and BR content between the control and empty TRV-infected seedlings. These results suggest that silencing the *SlSERK3B* gene may have an effect on BR synthesis as well as BR signaling.

### 2.3. SlSERK3B Gene Silencing Inhibits the Growth and Development of Tomato Seedlings

**Figure 3:**
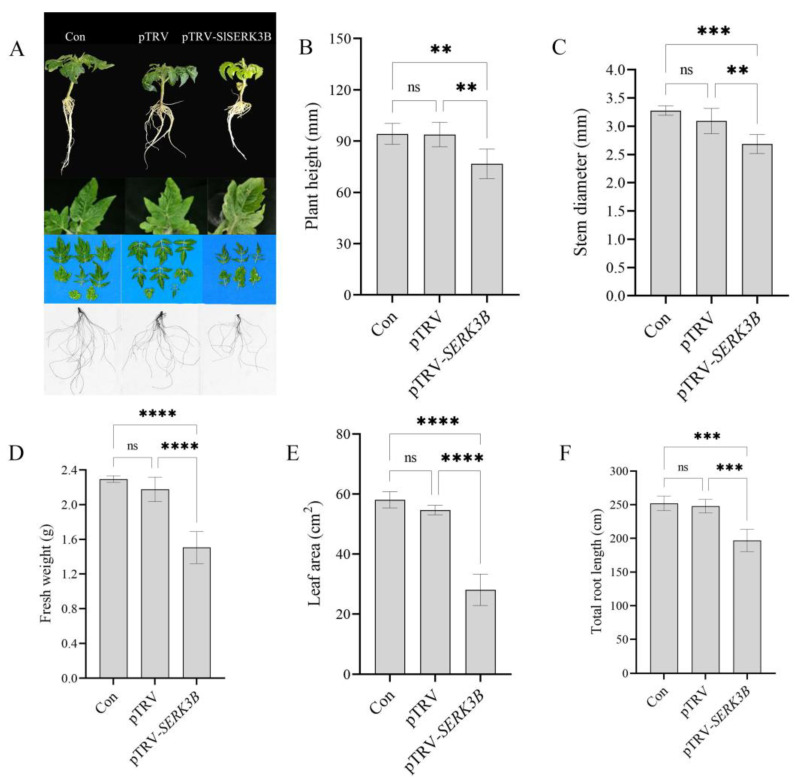
Effect of *SlSERK3B* gene silencing on the growth and development of tomato seedlings. (**A**) Photos of phenotype, leaf area, and total root length of control, empty TRV-infected and *SlSERK3B*-infected seedlings; (**B**) plant height; (**C**) stem diameter; (**D**) fresh weight; (**E**) leaf area; (**F**) total root length. Bars are means ± SD of three biological replicates. Asterisks indicate significant differences between control, empty TRV-infected, and *SlSERK3B*-infected seedlings (** *p* ≤ 0.01, *** *p* ≤ 0.001, **** *p* ≤ 0.0001, ns: no significant difference).

Compared with the control and empty TRV-infected seedlings, TRV-*SlSERK3B*-infected seedlings became shorter, and the leaves became curled and slightly yellowed (Figure 3A). Meanwhile, there was no significant difference in plant height, stem diameter, fresh weight, leaf area, and total root length between the control and empty TRV-infected seedlings. However, compared with the control and empty TRV-infected seedlings, the plant height of *SlSERK3B*-infected seedlings was decreased by 18.6% and 18.4%, respectively (Figure 3B). Additionally, compared with the control and empty TRV-infected seedlings, the stem diameter of *SlSERK3B*-infected seedlings was significantly decreased (Figure 3C). Similarly, the fresh were 34.1% lower and 30.7% lower in *SlSERK3B*-infected seedlings than in the control and empty TRV-infected seedlings, respectively (Figure 3D). Compared with the control and empty TRV-infected seedlings, the leaf area of *SlSERK3B*-infected seedlings was decreased by 51.6% and 48.6%, respectively (Figure 3E). Moreover, the total root length of *SlSERK3B*-infected seedlings was significantly higher than that of the control and empty TRV-infected seedlings (Figure 3F). Therefore, the *SlSERK3B* gene may significantly regulate tomato seedling growth.

### 2.4. SlSERK3B Gene Silencing Reduces Chlorophyll and Carotenoid Content

**Figure 4:**
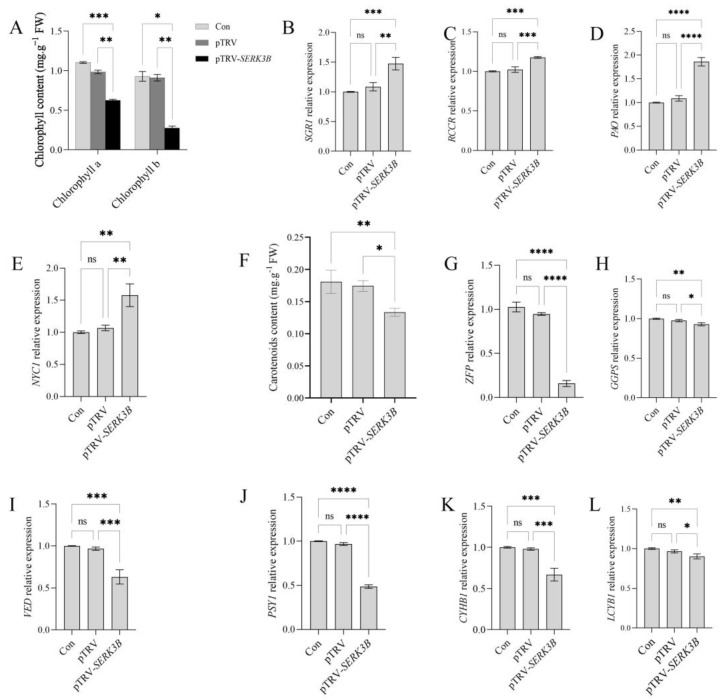
Effects of silencing the *SlSERK3B* gene on photosynthetic pigment content and related gene expression in tomato seedlings. (**A**) Chlorophyll a and b content; (**B**–**E**) expression levels of genes related to chlorophyll degradation. (**F**) Carotenoid content. (**G**–**L**) Expression levels of genes related to carotenoid synthesis. Expression values were normalized to the control for each gene. Bars are means ± SD of three biological replicates. Asterisks indicate significant differences between control seedlings, empty TRV-infected seedlings and TRV-*SlSERK3B*-infected seedlings (* *p* ≤ 0.05, ** *p* ≤ 0.01, *** *p* ≤ 0.001, **** *p* ≤ 0.0001, ns: no significant difference).

Compared with the control and empty TRV-infected seedlings, chlorophyll a and b levels were significantly reduced in *SlSERK3B*-infected seedlings (Figure 4A). Meanwhile, the expression levels of the chlorophyll-degrading genes *SlSGR1*, *SlRCCR*, *SlPAO*, *SlNYC1* and were significantly increased in the silenced plants (Figure 4B–E). In addition, carotenoid levels as well as the expression of carotenoid synthesis genes *SlPSY1*, *SlGGPS*, *SlLCYB1*, *SlCYHB1*, *SlVDE* and *SlZFP* were significantly reduced in *SlSERK3B*-infected seedlings compared to the control and empty TRV-infected seedlings (Figure 4F–L). There was no significant difference between the control and empty TRV-infected seedlings. Thus, silencing the *SlSERK3B* gene resulted in increased chlorophyll degradation and decreased carotenoid synthesis, thereby reducing both chlorophyll and carotenoid content.

### 2.5. SlSERK3B Gene Silencing Reduces Chlorophyll Fluorescence and Photosynthetic Parameters in Tomato Seedlings

**Figure 5:**
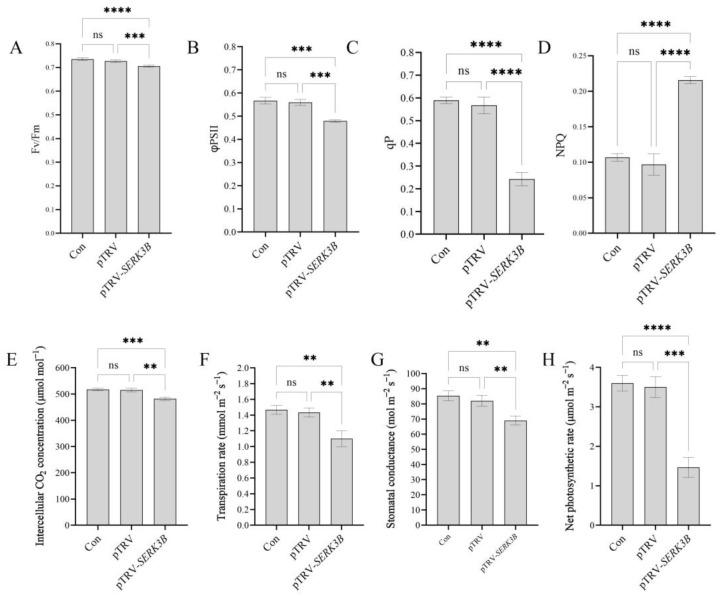
Effect of *SlSERK3B* gene silencing on chlorophyll fluorescence parameters and photosynthetic parameters in tomato seedlings. (**A**) Fv/Fm values, (**B**) ϕPS II values, (**C**) qP values, (**D**) NPQ values, and (**E**) Ci values. (**F**) Tr values. (**G**) Gs values. (**H**) Pn values. Bars are means ± SD of three biological replicates. Asterisks indicate significant differences between control seedlings, empty TRV-infected seedlings and TRV-*SlSERK3B*-infected seedlings (** *p* ≤ 0.01, *** *p* ≤ 0.001, **** *p* ≤ 0.0001, ns: no significant difference).

The Fv/Fm of the seedlings infected with TRV-*SlSERK3B* was significantly decreased compared to the control and empty TRV-infected seedlings (Figure 5A). Compared with the control and empty TRV-infected seedlings, the ϕPSII of seedlings infected with TRV-*SlSERK3B* decreased by 15.5% and 14.3%, respectively (Figure 5B). The qP was 58.9% lower and 57.3% lower in *SlSERK3B*-infected seedlings than in the control and empty TRV-infected seedlings, respectively (Figure 5C). Meanwhile, the non-photochemical quenching (NPQ) of TRV-*SlSERK3B*-infected seedlings was increased by 101.9% and 122.7%, respectively, compared to the control seedlings and empty TRV-infected seedlings (Figure 5D). This further suggests that the *SlSERK3B* gene may inhibit the growth and development in tomato seedlings by reducing chlorophyll fluorescence parameters.

Compared with the control and empty TRV-infected seedlings, the CO_2_ concentration of *SlSERK3B*-infected seedlings was significantly reduced (Figure 5E). The transpiration rate was 25.2% lower and 23.1% lower in *SlSERK3B*-infected seedlings than in the control and empty TRV-infected seedlings, respectively (Figure 5F). Meanwhile, compared with the control and empty TRV-infected seedlings, the *SlSERK3B*-infected seedlings showed significantly lower stomatal conductance (Figure 5G). In addition, the net photosynthetic rate decreased more in TRV-*SlSERK3B*-infected seedlings, accounting for 40.8% and 42.0% of the control and TRV-infected seedlings, respectively (Figure 5H). However, there was no significant difference in chlorophyll fluorescence and photosynthetic parameters between the control and TRV-infected seedlings. These results suggest that the *SlSERK3B* gene may play an important role in the photosynthesis of tomato seedlings.

## 3. Discussion

BR studies in growth and development cover a wide range of plant species. Of these, the roles of BR in plant growth and development have been most extensively studied in *Arabidopsis thaliana* [23]. BR was shown to be involved in jasmonate (JA) signaling and to negatively regulate JA-inhibited root growth [24]. Meanwhile, BRs have been shown to regulate root growth. In the present study, exogenous EBR treatment significantly increased plant height, stem diameter, as well as fresh weight in tomato seedlings. Another study reported that increasing endogenous BR levels improved tomato plant growth and produced a distinctive phenotype characterized by elongated and compact structures [25]. Similarly, EBR treatment showed a better appearance of the above- and below-ground parts of tomato seedlings in our study. Therefore, based on previous studies and our study, it can be demonstrated that BR has a positive effect on the growth of both the above-ground parts and roots of plant. However, the pathway by which BR promotes plant growth needs further investigation.

BR regulate plant development through a signal transduction pathway involving the *BRI1* and *BAK1* transmembrane receptor kinases [8]. We speculate that the promotion role of BR in tomato seedling growth and development may be related to the BR signaling pathway. In our study, EBR treatment significantly increased the expression of the BR receptor gene *BRI1*, the co-receptor gene *SERK3A* and its homolog *SERK3B*, and the transcription factor *BZR1*. The BR signaling pathway regulated primary root development and drought stress response by suppressing the expression of *PLT1* and *PLT2* in *A. thaliana* [26]. This further suggests that key genes in the BR signaling pathway may be involved in the promotion of plant growth and development.

BAK1 in the BR signaling pathway has been reported to mediate light-induced phosphorylation and catalase activation to regulate growth and development in *A*. *thaliana* [27]. Another study demonstrated the unique regulatory role of BR signaling during tomato fruit development, and it was suggested that the *SlSERK3B* gene might affect plant growth and development. In our study, the *SlSERK3B* gene was effectively silenced in the roots and leaves of tomato seedlings. However, the expression levels of *SlSERK1* and *SlSERK3A*, which are homologous to *SlSERK3B*, did not change significantly, indicating that only *SlSERK3B* was silenced. Meanwhile, *SlSERK3B*-silenced seedlings showed a significant increase in both endogenous BL levels and expression levels of BR synthesis genes. This may be due to the disruption of BR signaling and the presence of a negative feedback regulatory mechanism, leading to an increase in endogenous BL levels. The plant height, stem diameter, fresh weight, leaf area, and total root length in TRV-*SlSERK3B*-infected seedlings were significantly reduced. Meanwhile, TRV-*SlSERK3B*-infected seedlings became shorter, and the leaves became curled and slightly yellowed. In a similar way, previous study has shown that brassinosteroid-insensitive dwarf mutants of Arabidopsis accumulated brassinosteroids [28]. Therefore, it can be concluded that the *SlSERK3B* gene might play an important role in growth and development in tomato seedlings. Similarly, study has shown that co-silencing of *SlSERK3A* and *SlSERK3B* resulted in spontaneous necrotic lesions and reduced sensitivity to exogenous BR treatment [9]. This further suggests that *SlSERK3B* gene silencing hinders BR signaling and is detrimental to plant growth. However, more research is needed to understand how the *SlSERK3B* gene affects plant growth and development.

BRs are plant steroid hormones known to positively regulate photosynthesis [29]. BR has previously been shown to promote photosynthesis and growth in cucumber by positively regulating the synthesis and activation of several photosynthetic enzymes, including Rubisco [30]. Another study showed that endogenous BR regulated photosynthetic capacity mainly by activating the Calvin cycle enzymes [31]. We therefore speculate that *SlSERK3B* might affect the growth and development in tomato seedlings, possibly in relation to photosynthesis. In addition, chlorophyll biosynthetic enzymes are regulated by BR signaling in terms of gene expression [32]. It is well known that photosynthesis in plants begins with the absorption of light energy by photosynthetic pigments, and the photosynthetic capacity can be indirectly reflected by the content of photosynthetic pigments in plant leaves [33]. Chlorophyll content is a key indicator of photosynthetic capacity [34]. Carotenoids are ubiquitous and important pigments in photosynthesis. In our study, the levels of chlorophyll a, chlorophyll b and carotenoids were significantly reduced in *SlSERK3B*-infected seedlings. At the same time, the expression levels of chlorophyll-degrading genes were significantly increased, and carotenoid-synthesising genes were significantly decreased in the silenced plants. Thus, the *SlSERK3B* gene may have an effect on the photosynthetic capacity through the regulation of the synthesis and degradation of photosynthetic pigments, which in turn affects plant growth and development. Therefore, the relationship between *SlSERK3B* and plant photosynthetic capacity may be an important potential future research direction.

Chlorophyll fluorescence has been the main basis for studies of photosynthetic regulation and plant responses to the environment due to its sensitivity, convenience and non-invasiveness [35]. In this study, Fv/Fm, ϕPSII and qP levels in TRV-*SlSERK3B*-infected seedlings were significantly reduced, but NPQ was significantly increased. Similarly, a recent study showed that the *altered brassinolide sensitivity 1 (abs1)* mutant had significantly lower Fv/Fm, ϕPSII, and qP but significantly higher NPQ compared with wild-type leaves [2]. Changes in stomatal conductance to CO_2_ lead to changes in intercellular CO_2_ concentration (Ci), which affects photosynthetic rate [36]. In this study, intercellular CO_2_ concentration, transpiration rate, stomatal conductance and net photosynthetic rate were significantly reduced in *SlSERK3B*-infected seedlings. Thus, silencing the *SlSERK3B* gene might reduce electron transfer activity and photochemical efficiency, prevent charge accumulation and increase non-photochemical losses. Thus, silencing of the *SlSERK3B* gene would potentially impede BR signaling. At the same time, the absence of BR signaling in turn might have an impact on plant photosynthesis.

## 4. Materials and Methods

### 4.1. Plant Materials, Treatments and Growth Conditions

The seedlings of tomato (*Lycopersicum esculentum* L. ‘Micro-Tom’) were used as plant materials in this study. The seeds were surface disinfected with 1% of NaClO and transferred to 1/2 Hogland solution for 7 d after germination and then cultivated in Hogland solution for another 21 d. Similarly grown tomato seedlings were then collected and treated. EBR was added Hoagland nutrient solution to form a 0.05 μmol L^−1^ treatment solution, and the seedlings treated with the Hogland solution with no extra compounds added were served as the control. The concentration of EBR was selected based on pretesting. Each treatment consisted of three replicates, each consisting of sixty seedlings, and plant seedlings from each replicate were harvested separately for the following experimental analyses.

The experimental environment was kept at 16 h light (250 µmol m^−2^ s^−1^ photon irradiance) at 24 °C and 8 h dark at 20 °C in 60% relative humidity [37].

### 4.2. Quantitative Real-Time RT-PCR Analysis

Total RNA was extracted from the samples using TRIzol reagent (Invitrogen, Carlsbad, CA, USA), taking advantage of the FastQuant First Strand cDNA Synthesis Kit (Tiainen, Beijing, China) to synthesize cDNA (The reaction system was 2 µL RNA, 2 µL 5× Evo M-MLV Reagent Premix and 6 µL ddH_2_O). The cDNA concentration was diluted to 500 ng. µL^−1^. These reactions were carried out under the following conditions: 37 °C for 15 min, 85 °C for 5 s, and finally ending at 4 °C. LightCycler 480 Real-Time PCR System (Roche Applied Science, Penzberg, Germany) and SYBR Green Premix Pro Taq HS Premix kit were used for qRT-PCR. The reaction system was 2×SYBR Green Pro Taq HS Premix 10 µL, primer (the concentration was 10 μmol L^−1^) F 0.4 μL, primer R 0.4 μL, cDNA 2 μL, and ddH_2_O 7.2 μL. The primers used in the qRT-PCR were designed using Primer 5.0, and their specificity was confirmed by melting curve analysis. PCR cycling was performed as follows: 2 min at 95 °C followed by 39 rounds of 5 s at 95 °C, 30 s at the optimal annealing temperature and finally, 1 cycle of 5 s at 65 °C. A melting curve (65–95 °C; at increments of 0.5 °C) was generated to verify the specificity of primer amplification. Three replicates of each tissue sample were used to monitor possible sampling and experimental errors. The efficiency of each primer pair was determined by generating a standard curve using BR sequence dilutions of the cDNA. The ct values were within the linear amplification range to ensure the reliability of the data. The internal reference was Actin (NM-001247874.2), as shown in Table S1. The qRT-PCR data were analyzed using the 2^−ΔΔCt^ calculation method [38].

### 4.3. Determination of Brassinolide Content

The tomato leaves and roots (1 g) were taken and ground thoroughly with liquid nitrogen, then samples were transferred to a 10 mL centrifuge tube, and 5 mL of pre-cooled 80% chromatographic methanol was added. The mixture was shaken overnight at 4 °C and then centrifuged, and the supernatant was added to a centrifuge tube containing 10 mL of an 80% methanol solution. The 2 mL of supernatant was added to a brown tube and evaporated by spinning at 1300× *g* for 4–5 h at 38 °C on a vacuum centrifuge concentrator until completely dry. The dry extract was redissolved in 1 mL of 50% methanol. To ensure the accuracy of the measurements, [^2^H_3_] BL (2 ng) was added as an internal standard at this stage. The dissolved solution was filtered by a 0.22 μm microporous membrane, and the extract solution was collected for further analysis. The BL content was determined by means of liquid chromatography-mass spectrometry. The content of endogenous BL in tomato leaves and roots was determined by quaternary gradient ultra-fast liquid chromatography using a Waters Acquity ARC 600-2998 (Waters Corporation, Milford, MA, USA) equipped with the Symmetry-C18 column (2.1 mm × 50 mm, 1.8 μm, Agilent). The sample injection volume was 5 μL. The mobile phase consisted of a binary eluent solvent system of 0.1% formic acid (solvent A) and acetonitrile (solvent B), with a flow rate of 0.3 mL min^−1^; the column temperature was 30 °C. Gradient elution conditions: 0.0–1.0 min, 5% A; 1.0–2.0 min, 5–30% A; 2–3 min, 30–45% A; 3–7 min, 45–95% A; 12–13 min, 95–5% A; 13–15 min, 5% A. Mass spectrometry conditions: High-performance liquid chromatography-tandem mass spectrometry (HPLC-MS/MS) electrospray ionization source was used with the reaction monitoring (Srm) ion source mode selected as electrospray ionization and the ion source polarity as ESI+; ion source temperature: 350 °C; gas flow rate N_2_: 1.0 L h^−1^; Ar: 0 mL min^−1^; capillary voltage: 3.00 kV; desolventization temperature: 650 °C; cone pore gas flow rate: 0 L h^−1^; hexapod lens voltage: 0 V.

### 4.4. Construction of VIGS Vectors

The specific fragment of *SlSERK3B* gene silencing was obtained by PCR amplification using cDNA as template. The reaction system was 25 μL KOD DNA polymerase, 2 μL primer F, 2 μL primer R, 2 μL CDNA, 19 μL ddH2O. The cycle parameters were: initial denaturation: 98 °C × 2 min; denaturation: 98 °C × 10 s, annealing 58 °C × 10 s; extension: 68 °C × 19 s, 35 cycles; final extension: 72 °C × 2 min; hold: 4 °C. The target sequence of the *SlSERK3B* gene was amplified with specific primers, forward 5′-agaaggcctccatggggatccTGGACAACTCAAGAGGTTCTCCTT-3′ and reverse 5′-gagacgcgtgagctcggtaccACACTTCCATTTGCCATATATGGA-3′, which were designed by Primer 5.0 software. Then, the pTRV2 plasmid was double digested with BamHI and KpnI, and the target fragment was ligated to the empty vector. Then, the ligated vector was transferred into *Escherichia coli* (the name of the strain is DH5α), and positive colonies were obtained. The sequenced and validated vectors pTRV1-pTRV2 and pTRV2-*SlSERK3B* were transferred into *Agrobacterium tumefaciens* (the name of the strain is GV3101) as well. TRV1 and TRV2 were from our laboratory [39].

### 4.5. Agroinfiltration

A single colony of pTRV2-*SlSERK3B*, pTRV1 and pTRV2 was added to 2–3 mL of liquid medium, respectively. The bacterial suspension (500 μL) was added to a new LB liquid medium containing 100 mmol L^−1^ MES and 20 µmol L^−1^ AS and shaken at 28 °C for 14–15 h until the bacterial solution was particularly turbid. After centrifugation of the bacterial fluid, the supernatant was removed; then, the precipitate was added to a sterile buffer (100 mmol L^−1^ MgCl2, 10 mmol L^−1^ MES, 200 µmol L^−1^ AS, pH 5.6), and the buffer concentration was adjusted to an OD_600_ of 1.2–2.0. Two bacterial strains were prepared, one containing the pTRV1 and pTRV2 vectors and the other containing the pTRV2-*SlSERK3B* vector. The two strains were mixed in a 1:1 ratio. The mixture was then shaken at 100 revolutions per minute at a temperature of 28 °C. The mixture was then incubated with the pTRV1 and pTRV2 vectors. This shaking and incubation process lasted for 3 h [40]. The mixture was injected into 2-week-old tomato cotyledons all abaxially with a sterile syringe. The tomato seedlings were grown for approximately one month (four-leaf stage), and the seedlings were measured for index and observed phenotypically under the same temperature and photoperiod conditions. Twenty tomato seedlings were injected for each virus-induced gene silencing (VIGS) construct.

### 4.6. Measurement of Morphological Indexes

Plant height was measured by a Vernier caliper at a straight-line distance from the stem base to the apex of the shoot apical meristem. Stem diameter was measured with a Vernier scale through the cross method at the stem base of the first leaf mark. Fresh weight was obtained by electronic scales. Leaf area index was detected by a leaf area scanner (YMJ-C, Zhejiang Top Co. LTD., Hangzhou, China) of leaves on the whole single plant. The treated seedlings were removed after the above-ground parts, the impurities on roots were carefully washed out by distilled water and the images were scanned by root scanner (STD4800, Toronto, ON, Canada), and then total root length of each plant was determined by root analysis software WinRHIZO 5.0 (Regent Instruments, Inc., Quebec City, QC, Canada). Mean values of plant height, stem diameter, fresh weight, leaf area, and total root length in each treatment were calculated by 6 seedlings.

### 4.7. Measurements of Photosynthetic Pigment Contents

Chlorophyll pigment content was measured according to the following procedure. Briefly, the leaves from the same position of each tomato seedling were cut into 0.2 cm pieces and thoroughly mixed. the mixed tomato leaves (about 0.15 g) were put into a tube containing 10 mL of 80% acetone. The test tubes were then placed at room temperature under dark conditions for 24 h. When the leaves turned white, the tube was fixed with 80% acetone to 25 mL, and the absorbance was measured at 665 nm, 649 nm, and 470 nm, respectively [33,41].

### 4.8. Measurements of Chlorophyll Fluorescence and Photosynthetic Parameters

The FMS-2 pulse-modulated fluorometer (Hansatech Instruments Ltd., Norfolk, UK) was used to measured chlorophyll fluorescence parameters of tomato leaves [42]. The maximum photochemical efficiency (Fv/Fm), actual photochemical efficiency (ϕPS II), non-photochemical quenching (NPQ), and photochemical quenching (qP) were obtained under photoadaptation. The net photosynthetic rate (Pn), stomatal conductance (Gs), transpiration rate (Tr), and intercellular carbon dioxide concentration (Ci) of the second parietal leaf were measured using the CIRAS-2 portable photosynthesis meter (PP Systems, Massachusett, USA).

### 4.9. Statistical Analysis

All experiments were conducted with at least three independent biological replicates, and all reported data were presented as mean ± standard deviation (SD). Statistical analysis was performed using SPSS statistical software 22.0 (SPPS Inc. Chicago, IL, USA). Multiple comparisons were performed with the Tukey’s test when one-way ANOVA showed a significant effect (* *p* ≤ 0.05, ** *p* ≤ 0.01, *** *p* ≤ 0.001, **** *p* ≤ 0.0001). Means ± SD was based on three replicates. Graphs were constructed using GraphPad prism 9.0.0 (GraphPad Software, San Diego, CA, USA).

## 5. Conclusions

In conclusion, EBR treatment significantly promoted the growth and development in tomato seedlings and upregulated the expression of key genes in the BR signaling pathway, especially *SlSERK3A* and *SlSERK3B*. In addition, silencing the *SlSERK3B* gene disrupted BR signaling and prevented normal plant growth. In the absence of BR signaling, photosynthesis was inhibited due to increased chlorophyll catabolism and decreased carotenoids synthesis, further inhibiting plant growth. Collectively, our study may provide a direction for the study of roles of BR signaling pathway-related genes in plant growth and development. However, whether other genes in BR pathway play the same role as the *SlSERK3B* gene requires further investigation.

## Data Availability

All data, tables, and figures in this manuscript are original.

## Supplementary Materials

The following supporting information can be downloaded at: https://www.mdpi.com/article/10.3390/ijms25021336/s1.
